# Association of anaemia in primary care patients with chronic kidney disease: cross sectional study of quality improvement in chronic kidney disease (QICKD) trial data

**DOI:** 10.1186/1471-2369-14-24

**Published:** 2013-01-25

**Authors:** Olga Dmitrieva, Simon de Lusignan, Iain C Macdougall, Hugh Gallagher, Charles Tomson, Kevin Harris, Terry Desombre, David Goldsmith

**Affiliations:** 1 Department of Health Care Management and Policy, University of Surrey, Guildford, Surrey, GU2 7XH, UK; 2 Renal Medicine, Cheyne Wing, King’s College Hospital, London, SE5 9RS, UK; 3 Epsom & St Helier University Hospitals NHS Trust SW Thames Renal Unit, St. Helier Hospital, Wrythe Lane, Carshalton, Surrey, SM5 1AA, UK; 4 Southmead Hospital, Southmead Road, Westbury-on-Trym, Bristol, BS10 5NB, UK; 5 University Hospitals of Leicester, Gwendolen Road, Leicester, LE5 4PW, UK; 6 Renal and Transplantation Department, Guy's Hospital, 6th Floor, Borough Wing, Great Maze Pond, London, SE1 9RT, UK

**Keywords:** Aspirin, Chronic, Anaemia, Data collection, Erythropoietin, Family practice, Iron-deficiency, Medical records systems, Computerized, Renal insufficiency chronic

## Abstract

**Background:**

Anaemia is a known risk factor for cardiovascular disease and treating anaemia in chronic kidney disease (CKD) may improve outcomes. However, little is known about the scope to improve primary care management of anaemia in CKD.

**Methods:**

An observational study (N = 1,099,292) with a nationally representative sample using anonymised routine primary care data from 127 Quality Improvement in CKD trial practices (ISRCTN5631023731). We explored variables associated with anaemia in CKD: eGFR, haemoglobin (Hb), mean corpuscular volume (MCV), iron status, cardiovascular comorbidities, and use of therapy which associated with gastrointestinal bleeding, oral iron and deprivation score. We developed a linear regression model to identify variables amenable to improved primary care management.

**Results:**

The prevalence of Stage 3–5 CKD was 6.76%. Hb was lower in CKD (13.2 g/dl) than without (13.7 g/dl). 22.2% of people with CKD had World Health Organization defined anaemia; 8.6% had Hb ≤ 11 g/dl; 3% Hb ≤ 10 g/dl; and 1% Hb ≤ 9 g/dl. Normocytic anaemia was present in 80.5% with Hb ≤ 11; 72.7% with Hb ≤ 10 g/dl; and 67.6% with Hb ≤ 9 g/dl; microcytic anaemia in 13.4% with Hb ≤ 11 g/dl; 20.8% with Hb ≤ 10 g/dl; and 24.9% where Hb ≤ 9 g/dl. 82.7% of people with microcytic and 58.8% with normocytic anaemia (Hb ≤ 11 g/dl) had a low ferritin (<100ug/mL). Hypertension (67.2% vs. 54%) and diabetes (30.7% vs. 15.4%) were more prevalent in CKD and anaemia; 61% had been prescribed aspirin; 73% non-steroidal anti-inflammatory drugs (NSAIDs); 14.1% warfarin 12.4% clopidogrel; and 53.1% aspirin and NSAID. 56.3% of people with CKD and anaemia had been prescribed oral iron. The main limitations of the study are that routine data are inevitably incomplete and definitions of anaemia have not been standardised.

**Conclusions:**

Medication review is needed in people with CKD and anaemia prior to considering erythropoietin or parenteral iron. Iron stores may be depleted in over >60% of people with normocytic anaemia. Prescribing oral iron has not corrected anaemia.

## Background

Anaemia is an independent risk marker for cardiovascular morbidity and mortality and it is a common complication in patients with chronic kidney disease (CKD) [1-3]. CKD is divided into stages 1 to 5, with stage 3 sub-divided into two sub-stages, 3A and 3B. Haemoglobin (Hb) levels decline as renal function deteriorates [4]. The prevalence of anaemia (Hb less than 12 g/dl in men and 11 g/dl in women) is 1% in people with stage 3 CKD; 9% in stage 4; and 33% in stage 5 CKD [5]. Anaemia affects over two-thirds (68%) of people starting dialysis [6]; and 49.6% of men and 51.2% of women in stage 4 and 5 CKD not referred to renal specialists are anaemic [7]. Anaemia in patients with CKD and end stage renal disease (ESRD) has been associated with fatigue and reduced exercise capacity; poorer quality of life, higher incidences of myocardial infarction, congestive heart failure, and increased left ventricular mass index (LVMI) [8-12]. Diabetes mellitus is also associated with a doubling of the prevalence of anaemia in CKD [13]; there are also concerns that angiotensin converting enzyme inhibitors (ACE-I) are associated with anaemia [14].

Patients with CKD have a high cardiovascular risk and effective anaemia management may improve outcomes [15-17]. Partial correction of anaemia in CKD patients has been associated with reduced LVMI and left ventricular hypertrophy [18,19], improved cardiovascular outcomes [20], lower rate of transfusions [21], improved quality of life [9], and, delayed renal failure progression in predialysis nondiabetic patients [22] and improved renal function in patients with severe heart failure [23]. In the UK the National Institute for Health and Clinical Excellence (NICE) recommends evaluation of possible causes of anaemia where Hb ≤ 11 g/dl and treatment with intravenous iron and erthyropoiesis-stimulating agents (ESA) to maintain Hb in the range 10-12 g/dl [24,25]. Anaemia treatment with ESA has been shown to improve clinical outcomes in non-CKD patients with CVD [23,26,27]. However, in CKD ESA use has failed to show a positive effects on CVD mortality and is instead associated with some negative clinical outcomes especially where Hb is corrected to over 12 g/dl [3,28-30]. The U.S. Food and Drug Administration (FDA) recommends to consider starting ESA treatment only when the Hb level is less than 10 g/dl for non-dialysis people with the CKD and anaemia [31].

Despite advances in the use of ESA for CKD patients, there is low uptake of this therapy [16]. Although it is suggested up to 10% of patients in Primary Care in UK have CKD [15] and up to 6% of CKD patients have anaemia [17] little is known about the aetiology, iron store status, and the extent to which family physicians may have tried to treat anaemia in practice.

We carried out this cross-sectional study to report the prevalence of anaemia in CKD, examine its association with cardiovascular diseases, and assess whether practitioners had attempted to treat the patients with oral iron.

## Methods

We used data from the Quality Improvement in Chronic Kidney Disease (QICKD - ISRCTN5631023731) trial [32,33]. The QICKD trial was conducted in 127 practices drawn from localities across England and contains extracts from the records of all registered patients within these practices, a total of 1,099,296 people. The population profile approximates to the national average in the 2001 Census with a small excess of working age people and slightly fewer older people aged 60 to 75 years (Additional file 1: Figure S1). The QICKD trial practices are usual general practices with routine data expected to be similar to those found in other practices. These data were collected at the trial mid-point between December 2009 and July 2010 using well established methods [34,35]. Our dataset included demographic details: age, gender, ethnicity and deprivation score. The latter used the index of multiple deprivation (IMD) [36]. Deprivation is a concept that overlaps with, but is not synonymous with poverty. IMD is calculated across the UK based on seven constituent parts to allow comparisons between areas. The seven domains of deprivation are: (1) income, (2) employment, (3) health deprivation and disability, (4) education skills and training, (5) barriers to housing and services, (6) crime and (7) the living environment. IMD is divided into deciles of equal sizes, where the first decile (IMD ≤5.63) is the least deprived and decile ten (IMD ≥ 45.33) the most deprived.

We defined cases of stages 3 to 5 CKD by the estimated glomerular filtration rate (eGFR), taking into account the requirement of three month period of chronicity for formal diagnosis. Wherever available we used laboratory calculated eGFR because it is subject to a national calibration scheme [37]. We describe people with eGFR ≥90 ml/min/1.73 m^2^ as having a normal eGFR, those with eGFR ≥60 ml/min/1.73 m^2^ and <90 ml/min/1.73 m^2^ as having “mildly impaired renal function.” We subdivide those with an eGFR <60 ml/min/1.73 m^2^ into stages 3A, 3B, 4 and 5 using conventional eGFR ranges [38]. Where we use the term “CKD” alone we mean stage 3 to 5 CKD. We did not include diagnostic codes for CKD as these under-report the prevalence of CKD [39].

We extracted a dataset that included Hb, mean corpuscular volume (MCV) and common potential causes of anaemia so that we could differentiate between renal and other causes of anaemia. We defined anaemia as Hb ≤ 11 g/dl in line with NICE guidance [24].

We report the prevalence of Hb measurement among people with CKD and compare their Hb with those in people without CKD. We also explored what proportion of people had a recent measure of Hb, which we define as a recording within the last two years. We classify anaemia into micro-, normo- and macrocytic based on the MCV. Microcytic anaemia is defined as an MCV of <80 fl, normocytic as 100-80 fl, and macrocytic as >100 fl [40]. We also extracted ferritin values, as marker of iron stores. In CKD stage 3–4, iron deficiency was defined as ferritin <100ug/ml [41,42] as patients with depleted iron stores benefit from intravenous iron even where their MCV is normal; [43] though we also report ferritin < 15 ug/ml as this has been proposed as a lower limit of normal [44,45].

To explore the association between age and anaemia the prevalence of anaemia by eGFR category and age band we report the change using previously described groupings [46].

We explored any association between CVD, CKD and anaemia. We included in our definition of cardiovascular disease (CVD) diagnoses of heart failure (HF), ischaemic heart disease (IHD), stroke, transient ischaemic attack and cerebrovascular disease (CEVD), peripheral vascular disease (PVD), and hypertension (HT); and diabetes mellitus.

We investigated whether anaemic patients had been prescribed aspirin and non-steroidal anti-inflammatory drugs (NSAIDs), clopidogrel or warfarin. These are medications which are all associated with an increased risk of gastrointestinal bleeding.

Finally we looked at oral iron prescriptions in order to see if iron was being prescribed to correct anaemia and to assess whether its use was associated with correction of anaemia. As intravenous iron and ESA therapy is largely provided via specialist renal units, information about parenteral iron is not contained within the family practice computerised medical record.

The data used in the study were extracted from primary care computer systems and processed using our established method [34,47]. We analysed these data using SPSS (Statistical Package for Social Sciences, Version 18). We used simple descriptive statistics to report our findings; we used Pearson chi square to test whether proportions were significantly different reporting the probability and commenting if not significant (n.s.). We report differences in Hb between different subgroups using independent samples T-tests, reporting the mean difference and probability (p). We constructed a linear regression model to test the extent to which the variables reported in our model are predictors of any change in Hb.

We carried out a regression analysis initially testing each variable separately to explore any predictive effect on our outcome variable (Hb). We then grouped our best predictor variables into a single model reporting the unstandardised coefficient (*B*); it standard error (E); and significance (p). *B* represents the unit change in the outcome variable for either one unit of change, or the presence or absence of the predictor variable. E.g. eGFR has a *B* of 0.003, this means that for every 10 ml/min rise in eGFR Hb rises by 0.03 g/dl; *B* Stage 3 to 5 CKD is −0.482, implying that people with stage 3 to 5 CKD have an Hb 0.5 g/dl lower than those who do not. We did not include age, gender or ethnicity in our model as they are already included in the equation used to estimate renal function [48]. We did however include the use of ACE-I, ACE-I and hypertension, and hypertension alone in our model to explore any influence from a patient’s therapy. We then grouped our best predictive variables into a single model for which we additionally quote R2, the correlation coefficient which gives an effect size (i.e. to what extent the change seen can be ascribed to the variables in the model; an R2 of 0.11 implies that it contributes 11% of the change).

Ethical approval for the trial was given by the Oxford Research Ethics committee and is included in our clinical trial registration details (ISRCTN56023731) [49].

## Results

### Prevalence of CKD in the study population

The prevalence of CKD in the general population is 5.3% (n = 58,592); 6.76% (50,319) in people aged over 18 years. As renal function declines from normal to stage 3B CKD the mean age of the people gets older with each declining stage of CKD; those with stage 4 and 5 CKD are slightly younger (See Additional file 2: Figure S2), possibly due to survival bias.

### Prevalence of micro- , normo-, and macrocytic anaemia in CKD patients

We found that 94% of people with CKD have had an Hb measurement at some time, with 70% measured within the last 2 years. The mean Hb value for patients without CKD was 13.71 g/dl (median 13.7 g/dl, SD 1.6), compared to 13.22 g/dl (median 13.3 g/dl, SD 1.6) in patients with CKD. Furthermore, prevalence of anaemia was increased in the higher CKD stages (Table 1). The overall prevalence of anaemia (Hb ≤ 11 g/dl) in people with CKD Stage 3–5 is 8.6% (n = 4,690). 3.0% (n = 1,648) have Hb ≤ 10 g/dl and 1% (n = 563) Hb ≤ 9 g/dl. 22.2% of people with CKD had anaemia as defined by the World Health Organization (WHO) (Hb <12 g/dl in women and <13 g/dl in men).

**Table 1:** Prevalence of anaemia in patients with CKD

**Class CKD**		Hb < 13 g/dl(M)*		**Hb > 11 g/dl**		Hb ≤ 11 g/dl**		Hb ≤ 10 g/dl***		**Hb ≤ 9 g/dl**	
		**Hb < 12 g/dl(F)**									
		**N**	**%**	**N**	**%**	**N**	**%**	**N**	**%**	**N**	**%**
eGFR > 90	F	11545	18.5%	61287	92.8%	4732	7.2%	1523	2.3%	536	0.8%
	M	3552	6.3%	54902	98.6%	790	1.4%	340	0.6%	142	0.3%
eGFR 60-89	F	16032	11.1%	132285	96.0%	5455	4.0%	1747	1.3%	612	0.4%
	M	7228	6.0%	106310	98.8%	1281	1.2%	541	0.5%	235	0.2%
Stage 3A	F	4542	14.3%	28205	94.5%	1654	5.5%	508	1.7%	173	0.6%
	M	2997	20.4%	12760	95.1%	657	4.9%	254	1.9%	101	0.8%
Stage 3B	F	2340	36.4%	5065	82.6%	1065	17.4%	329	5.4%	96	1.6%
	M	1616	48.7%	2654	83.5%	523	16.5%	207	6.5%	86	2.7%
Stage 4	F	663	59.8%	680	63.6%	389	36.4%	167	15.6%	35	3.3%
	M	553	71.3%	516	69.2%	230	30.8%	102	13.7%	38	5.1%
Stage 5	F	128	61.2%	108	55.1%	88	44.9%	43	21.9%	16	8.2%
	M	176	81.5%	123	59.4%	84	40.6%	38	18.4%	18	8.7%
CKD stage 3-5	F	7673	19.4%	34058	91.4%	3196	8.6%	1047	2.8%	320	0.9%
	M	5342	28.1%	16053	91.5%	1494	8.5%	601	3.4%	243	1.4%
Total CKD 3-5		13015	22.2%	50111	91.4%	4690	8.6%	1648	3.0%	563	1.0%

*WHO anaemia definition. **NICE – UK guidelines ***FDA recommended level for anaemia correction.

**Figure 1:**
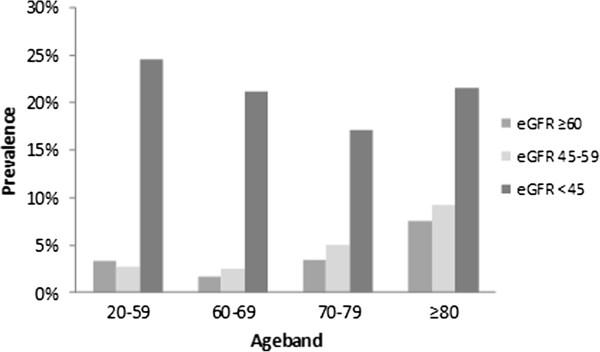
Age-specific prevalence of anaemia in CKD.

We observed in our data that anaemia in CKD is age-independent when eGFR <45 ml/min per 1.73 m2 as also found in a recent large US national cross-sectional study [46]. Anaemia prevalence increases as eGFR prevalence rises in all age groups (Figure 1). Anaemia was more common in older patients then in young patients with moderately decreased eGFR (Figure 1).

**Figure 2:**
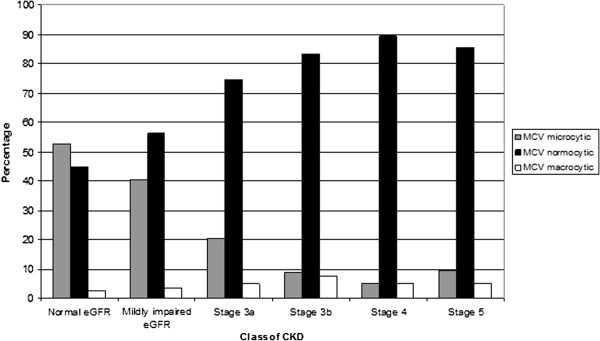
Micro-, normo-, and macrocytic anaemia for each stage CKD.

Normocytic anaemia is the most common form of anaemia in patients with CKD (Figure 2). 80.5% (n = 3,744) of patients with CKD and Hb ≤ 11 g/dl have normocytic anaemia. The proportion falls to three quarters (72.7%, n = 1,057) for Hb ≤ 10 g/dl and to two thirds (67.6%, n = 375) where Hb ≤ 9 g/dl. As Hb falls so the proportion of people with microcytic anaemia increases: from 13.4% (n = 625) where Hb ≤ 11 g/dl, to 20.8% (n = 302) where Hb ≤ 10 g/dl, to a quarter of those with Hb ≤ 9 g/dl (n = 138). The proportion of macrocytic anaemia only slightly rises as Hb falls in CKD: 6.0% (n = 280) of people with Hb ≤ 11 g/dl rising to 7.6% (n = 42) of people with Hb ≤ 9 g/dl (Table 2).

**Table 2:** Normocytic anaemia is the most common in patients with CKD

**MCV range**		**Microcytic**		**Normocytic**		**Macrocytic**	
	**Hb (g/dl)**	**N**	**%**	**N**	**%**	**N**	**%**
Female	Hb > 11	810	2.4%	32267	95.1%	842	2.5%
	Hb ≤ 11	481	15.1%	2536	79.7%	164	5.2%
	Hb ≤ 10	230	24.8%	647	69.7%	51	5.5%
	Hb ≤ 9	103	32.5%	195	61.5%	19	6.0%
Male	Hb > 11	398	2.5%	14955	93.8%	589	3.7%
	Hb ≤ 11	144	9.8%	1208	82.3%	116	7.9%
	Hb ≤ 10	72	13.7%	410	77.9%	44	8.4%
	Hb ≤ 9	35	14.7%	180	75.6%	23	9.7%
Total	Hb > 11	1208	2.4%	47222	94.7%	1431	2.9%
	Hb ≤ 11	625	13.4%	3744	80.5%	280	6.0%
	Hb ≤ 10	302	20.8%	1057	72.7%	95	6.5%
	Hb ≤ 9	138	24.9%	375	67.6%	42	7.6%

### Prevalence of Iron deficiency anaemia in CKD patients

Approximately one third (31%, n = 18,157) of people with CKD have had their ferritin measured. In two-thirds (63.7%, n = 11,570) of these patients the ferritin is <100ug/mL and 2.7% (n = 1,569) had a ferritin level <15ug/mL; suggesting that they had reduced iron stores. Over three-quarters (82.7%) of people with CKD and microcytic anaemia (Hb ≤ 11 g/dl) have a ferritin less than 100 ug/mL (Chi-square, p = 0.017), compared with 58.8% of those with normocytic (Chi-square p < 0.001) and 45.5% of those with macrocytic anaemia (Chi-square p = 0.8, n.s.). Furthermore, 39% patients with CKD and microcytic anaemia have ferritin level below 15 ug/mL (Table 3).

**Table 3:** Low ferritin level in CKD patients is associated with microcytic anaemia

**Ferritin**	**Microcytic**		**Normocytic**		**Macrocytic**		**All**	
**ug/mL**	**n**	**%**	**n**	**%**	**n**	**%**	**n**	**%**
<15	171	39.0%	236	10.3%	4	2.3%	**411**	**14.1**
15-99	192	43.7%	1117	48.5%	76	43.2%	**1385**	**47.5**
100-199	36	8.2%	469	20.4%	34	19.3%	**539**	**18.5**
≥200	40	9.1%	480	20.9%	62	35.2%	**582**	**20.0**
**Total**	**439**	**100**%	**2302**	**100**%	**176**	**100**%	**2917**	**100.0**

### Anaemia as a risk factor for CVD in patients with CKD

Analysis of cardiovascular co-morbidities and diabetes showed that these conditions are more prevalent among people with anaemia and CKD than those with CKD and a normal Hb. The prevalence of ischaemic heart disease (IHD), heart failure (HF), stroke and transient ischaemic attack grouped as cerebrovascular disease (CEVD), peripheral vascular disease (PVD) and diabetes is approximately twice that of CKD patients without anaemia (Table 4). In addition, 67.2% of patients with CKD and anaemia have hypertension as compared with 54% patients with CKD only (Chi-square p < 0.001).

**Table 4:** Anaemia is associated with CVD in CKD patients

	***Population***		***CKD***			
			***Hb > 11 g/dl***		***Hb ≤ 11 g/dl***	
***Female***	**N**	**%**	**N**	**%**	**N**	**%**
HT	71811	16.4%	17591	51.7%	2140	67.0%
Diabetes	19909	4.6%	4299	12.6%	923	28.9%
IHD	12309	2.8%	4329	12.7%	715	22.4%
HF	3659	0.8%	1642	4.8%	409	12.8%
PVD	2642	0.6%	851	2.5%	161	5.0%
CVA	9903	2.3%	3114	9.1%	534	16.7%
***Male***						
HT	60758	14.2%	9458	58.9%	1014	67.9%
Diabetes	24322	5.7%	3427	21.4%	519	34.7%
IHD	19729	4.6%	4251	26.5%	561	37.6%
HF	3927	0.9%	1407	8.8%	273	18.3%
PVD	3925	0.9%	952	5.9%	165	11.0%
CVA	9754	2.3%	2129	13.3%	319	21.4%
***Total***						
HT	132569	15.4%	27049	54.0%	3154	67.2%
Diabetes	44231	5.1%	7726	15.4%	1442	30.7%
IHD	32038	3.7%	8580	17.1%	1276	27.2%
HF	7586	0.9%	3049	6.1%	682	14.5%
PVD	6567	0.8%	1803	3.6%	326	7.0%
CVA	19657	2.3%	5243	10.5%	853	18.2%

Test of proportion (Chi-square) for all rows (p < 0.001) in favour of an increased prevalence of anaemia with CKD.

### Drug prescription in patients with CKD and anaemia

Two-thirds of patients (62%) in our study receive one or more drugs causing anaemia in the last two years, 84.7% ever. Nearly half of people with CKD and anaemia have been prescribed aspirin and non-steroidal anti-inflammatory drugs (NSAID) (49.1%, n = 2,301 and 46.2%, n = 2166 respectively), 8.1% (n = 378) received clopidogrel and 10.3% (n = 485) warfarin in the last two years. Analysis of medication history showed that 61% (n = 2,862) of people with CKD and Hb ≤ 11 g/dl had taken aspirin, three-quarters (73%, n = 3,422) NSAID, 14.1% (n = 662) were taking warfarin and 12.4% (n = 581) clopidogrel. Furthermore, more than half (55.6%, n = 999) of patients with iron deficiency anaemia and CKD stage 3–5 were concurrently prescribed NSAIDs and aspirin (Table 5).

**Table 5:** Prescribing in patients with CKD and anaemia

	**CKD**		**CKD & anaemia**		**CKD, anaemia, ferritin < 100ug/ml**		**CKD, anaemia, ferritin < 15ug/mL**	
	**N**	**%**	**N**	**%**	**N**	**%**	**N**	**%**
Aspirin	25218	43.1%	2862	61.0%	1133	63.1%	225	54.7%
Clopidogrel	4149	7.1%	581	12.4%	231	12.9%	44	10.7%
Warfarin	5697	9.7%	662	14.1%	248	13.8%	40	9.7%
NSAID	40121	68.5%	3422	73.0%	1363	75.9%	304	74.0%
Aspirin + NSAID	21614	36.9%	2490	53.1%	999	55.6%	194	47.2%
Aspirin + Clopidogrel	3453	5.9%	491	10.5%	198	11.0%	36	8.8%
Aspirin + Warfar	3461	5.9%	412	8.8%	160	8.9%	21	5.1%
NSAID + Clopidogrel	3350	5.7%	497	10.6%	206	11.5%	39	9.5%
NSAID + Warfarin	4047	6.9%	482	10.3%	182	10.1%	29	7.1%
Aspirin + NSAID + Clopidogrel	3006	5.1%	454	9.7%	188	10.5%	34	8.3%
Aspirin + NSAID + Warfarin	2992	5.1%	367	7.8%	142	7.9%	21	5.1%
Aspirin + NSAID + Clopid + Warfarin	495	0.8%	73	1.6%	26	1.4%	4	1.0%

### Oral Iron prescription

Over half (62.6%) of people with anaemia in the general population had been prescribed oral iron therapy (Table 6) with prescriptions issued to a slightly lower proportion (56.3%) of people with anaemia and CKD. The commonest prescribed iron preparations were ferrous fumarate (322 mg bd) and ferrous sulphate (200 mg tid); with the prescriptions intended to provide 200 mg or 195 mg elemental iron, respectively; in line with current recommendations [50].

**Table 6:** Oral iron prescription in patients with anaemia

		**Anaemia**			**CKD & anaemia**			**CKD anaemia & ferritin < 100ug/mL**			**CKD anaemia & ferritin < 15ug/mL**		
**Gender**	**Therapy**	**N**	**%**	**Hb mean**	**N**	**%**	**Hb mean**	**N**	**%**	**Hb mean**	**N**	**%**	**Hb mean**
Female	None	7212	34.5%	10.32	1341	42.0%	10.31	444	33.0%	10.31	70	21.0%	9.94
	Oral Iron	13693	65.5%	10.09	1855	58.0%	10.04	902	67.0%	10.05	264	79.0%	9.92
Male	None	2248	51.4%	9.91	707	47.3%	10.07	138	30.7%	10.09	15	19.5%	9.51
	Oral Iron	2122	48.6%	9.89	787	52.7%	9.91	312	69.3%	9.81	62	80.5%	9.47
Total	None	9460	37.4%	10.22	2048	43.7%	10.23	582	32.4%	10.26	85	20.7%	9.86
	Oral Iron	15815	62.6%	10.07	2642	56.3%	10	1214	67.6%	9.99	326	79.3%	9.83

Two-thirds (67.6%) of people with anaemia and CKD and a low ferritin level were taking oral iron. The mean Hb in the iron treated group was 10.0 g/dl (n = 582) compared with 10.3 g/dl (n = 1214) in the non-iron treated (t-test p < 0.001).

### Regression analysis

Stage 3–5 CKD was associated with a reduction in Hb, 0.712 g/dL (SE 0.017 g/dL, p < 0.001, R^2^ = 0.9%). The regression analysis testing each variable separately showed a small but significant effect of eGFR and CKD on the outcome variable, haemoglobin (*B* of 0.003, SE 0.000, p < 0.001, R^2^ = 0.2%). Our best model included: CKD stages 3–5, significant proteinuria, heart failure, diabetes, hypertension, stroke, deprivation score and NSAID within last 2 years, R^2^ = 7.4% (p < 0.001). After adding other elements to the model the CKD remained a significant contributor to the final effect (Table 7); though overall the combined predictive effect of the model on anaemia is small (R^2^ = 7.7%). We excluded ACE-I from our final model. The unstandardised coefficient (*B*) was smaller than that for hypertension and the overall model performed less well with ACE-I and hypertension included.

**Table 7:** Multiple regression model, predictor variables effect on the outcome variable (haemoglobin level)

	**Dependent variable - Haemoglobin level**			
**Predictor variables**	**Unstandardised coefficients**		**Standardised coefficients**	
	***B***	**Std. Error**	**Beta**	**T**
(Constant)	14.146	0.021		682.905
CKD stages3-5	−0.712	0.017	−0.204*	−42.385
Significant proteinuria	−0.501	0.033	−0.068*	−15.064
Heart failure	−0.368	0.033	−0.051*	−11.255
Diabetes	−0.078	0.016	−0.023*	−4.94
Hypertension	−0.144	0.015	−0.043*	−9.315
CEBVD or Stroke	−0.236	0.025	−0.042*	−9.309
Deprivation score	−0.01	0.001	−0.086*	−19.138
NSAID within last 2 years	−0.132	0.015	−0.04*	−8.955

R^2^ for the model = 7.4% significance for the model <0.001 (*p < 0.001).

## Discussion

In this study we found that anaemia is common in CKD and usually normocytic. Anaemia in CKD is associated with a reduced ferritin in over half, suggesting depleted iron stores. Microcytic anaemia is less common, though over three-quarters of people with this type of anaemia have a reduced ferritin. All cardiovascular diseases are more prevalent among those with anaemia and CKD compared with those with a normal Hb and CKD. Over three-quarters of people with anaemia and CKD are on one or more medications which may exacerbate anaemia; over three quarters have been prescribed aspirin as some time, over two-thirds in the last two years. Nearly three quarters of anaemic people with CKD have been prescribed an NSAIDs. Three quarters of people with microcytic anaemia and over half of those with normocytic anaemia have been prescribed oral iron, however despite this their anaemia remains uncorrected. Stage 3–5 CKD and reduction in eGFR are weak but significant predictors of reduced haemoglobin.

The prevalence of anaemia increased with reduction of eGFR levels in all age groups and this observation corresponds, and appears to validate, in an independent sample findings in the National Health and Nutrition Examination Survey (NHANES) population [4,5]. However, this prevalence is less than half that found in the more targeted National Kidney Foundation Kidney Early Evaluation Program (KEEP) [51].

Stage 3–5 CKD is an independent predictor variable of reduced haemoglobin. The current focus of UK National guidance on anaemia management in CKD needs to be reviewed. We recommend a shift from early referral to specialist centres for administration of parenteral iron or ESA to more effective medication management in primary care, with improved access to parenteral iron. Family physicians should carefully balance the risk benefit ratio of prescribing aspirin in cardiovascular disease and of NSAIDs in people with CKD. The concurrent use of acid suppressant therapy may help reduce the risk of gastrointestinal blood loss.

Our findings are consistent with a systematic review and meta-analysis that showed little benefit from oral iron in people with CKD. Interestingly, the rise in Hb reported using parenteral iron (0.83 g/dL) is similar to the decline seen in people with CKD (0.72 g/dL) [52].

In USA the Third National Health & Nutrition Examination Survey Public health (NHANES III) reported a high prevalence of anaemia, and a low ferritin in people with heart failure and CKD, which is also compatible with our results [53]. Likewise, NHANES found 42.2% (95% confidence interval 28.3-56.0%) of people with eGFR <40 ml/min were anaemic compared with 34% in this study. Antihypertensive medication, including angiotensin-converting enzyme inhibitors are also associated with anaemia [14,54]. We included hypertension, not anti-hypertensive therapy in our regression model. It is possible that the small reduction in haemoglobin associated with hypertension might be related to therapy.

Our approach is limited by the inevitable incompleteness of the routine data, though their strengths and weaknesses are well known [55]. The associations reported in this paper do not prove or imply a causative link; and paradoxically agents that might cause gastrointestinal haemorrhage were not associated with greater degrees of iron deficiency.

A further difficulty is that definitions of anaemia are not completely standardised. The European guidelines define anaemia as <11 g/dl [56], which is marginally different from guidance in England. The World Health Organization (WHO) define anaemia as <12 g/dl in women and <13 g/dl in men [57]. Whilst prescription of oral iron was not associated with correction of anaemia; we cannot conclude that it is ineffective as we don’t know the pre-treatment Hb levels. Although generally 200 mg of elemental iron is the default prescription in UK family practice, we know that it is common practice in primary care to advise patients to reduce the iron dose if they experience gastrointestinal side effects. We also only have evidence a prescription was issued, and nothing about what was actually dispensed.

Serum ferritin <100 ng/mL is used in this paper as a surrogate for iron deficiency; this is an expert consensus, used in UK national guidance [58] though many normal individuals having levels beneath this threshold [59].

Prospective studies are needed to assess whether more effective anaemia management strategies might reduce the incidence of CVD in people with CKD. Tools and algorithms are needed to help family doctors asses the relative risk of stopping NSAIDs, aspirin and other therapy against the potential benefits of correcting anaemia.

## Conclusion

Anaemia is common among CKD patients in the UK and associated with cardiovascular diseases and prescription of drugs which may cause anaemia. Having stage 3–5 CKD is a predictor variable of a decline in haemoglobin. Primary care management should start with a careful medication review and assessment prior to referral for replacement of depleted iron stores with parenteral iron.

## Abbreviations

ACE-I: Angiotensin converting enzyme inhibitors; ARB: Angiotensin II receptor blockers; CEVD: Cerebrovascular disease; CKD: Chronic kidney disease; CVD: Cardiovascular disease; eGFR: Estimate of glomerular filtration rate; ESA: Erthyropoiesis-stimulating agents; FDA: Food and Drug Administration; Hb: Haemoglobin; HT: Hypertension; HF: Heart failure; IHD: Ischaemic heart disease; IMD: Index of multiple deprivation; LVMI: Left ventricular mass index; MCV: Mean corpuscular volume; NSAID: Non-steroidal anti-inflammatory drugs; NICE: National Institute for Health and Clinical Excellence; NHANES III: Third National Health & Nutrition Examination Survey Public health; PVD: Peripheral vascular disease; SPSS: Statistical Package for Social Sciences Version 18; QICKD: Quality Improvement in Chronic Kidney Disease; WHO: World Health Organization.

## Competing interests

OD: None. SdeL: Involved in development of the pay-for-performance indicator for UK practice; lead author for Department of Health Frequently Asked Questions about chronic kidney disease. http://www.nhsemployers.org/SiteCollectionDocuments/Chronic_kidney_disease_FAQs%20-%20ja040711.pdf. IMacD: Professor Macdougall has received consultancy and lecture honoraria from several companies involved in the manufacture of ESAs and IV iron products, and is a current Work Group member of the KDIGO Anemia Guidelines Group. HG: None. CT: None. KH: Kevin Harris has received funding from several pharmaceutical companies, Edith Murphy Foundation 2007–2010: Quality Improvement in CKD due to diabetes and LNR CLAHRC for Prevention of Chronic Disease and its Associated Co-Morbidity theme. TD: None. DG: Honoraria and Speaker Fees from Takeda, Roche, Sandoz, Amgen.

## Authors’ contributions

OD, SdeL, ICM and DG conceived and designed the study; OD wrote the first draft of the manuscript, which SDEL developed. OD and SDEL performed the analysis. OD, SdeL, ICM, HG, CT, KH, TD and DG. All authors contributed to editing the paper, and approved the final version of the paper.

## Pre-publication history

The pre-publication history for this paper can be accessed here:

http://www.biomedcentral.com/1471-2369/14/24/prepub

## Supplementary Material

Additional file 1 **Figure S1. **Age sex profile of study population compared to census data. Click here for file

Additional file 2 **Figure S2. **Patients by stage CKD, age and sex. Click here for file
