# Supplementary figures and images for: Association of anaemia in primary care patients with chronic kidney disease: cross sectional study of quality improvement in chronic kidney disease (QICKD) trial data
Source: BMC Nephrol. 2013 Jan 25;14:24. doi: 10.1186/1471-2369-14-24 (PMC3626717; doi:10.1186/1471-2369-14-24)

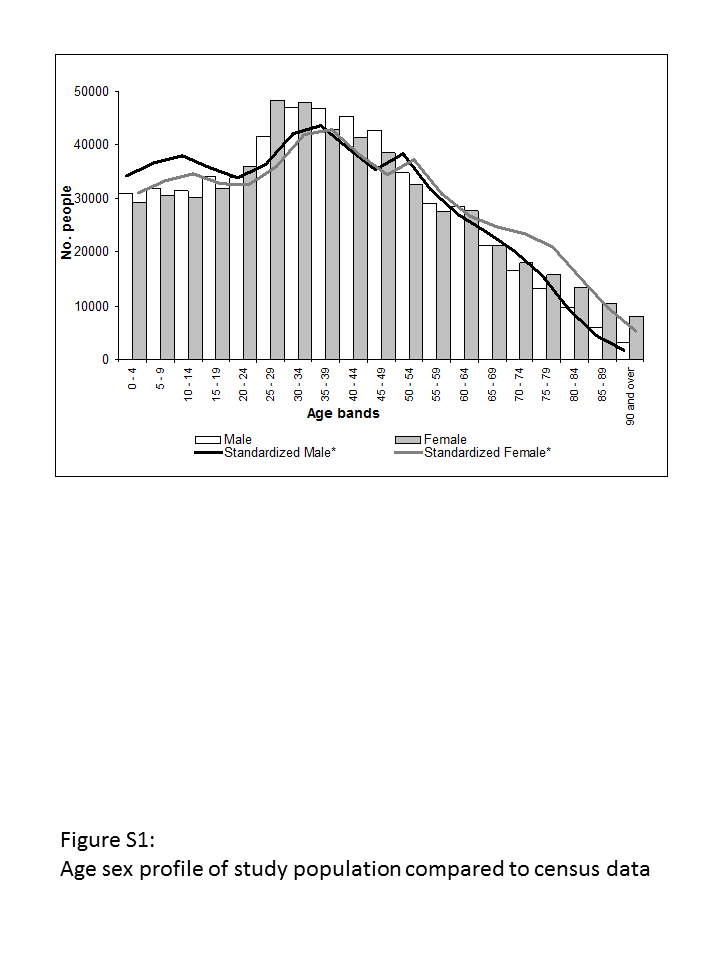

Supplement: Additional file 1 — Figure S1. Age sex profile of study population compared to census data. [file 1471-2369-14-24-S1.tiff]

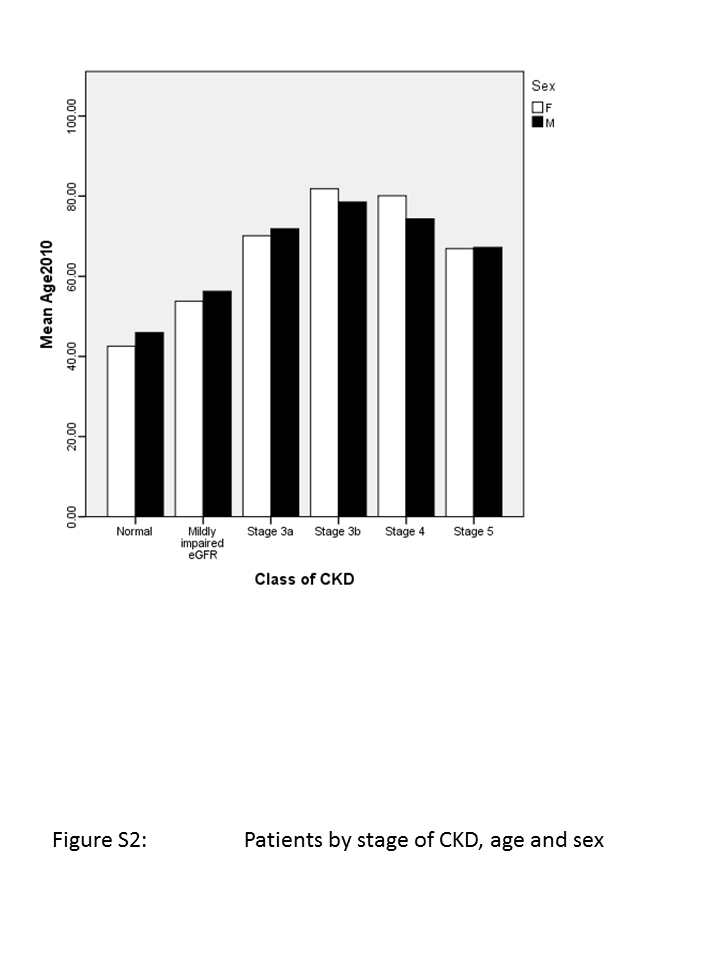

Supplement: Additional file 2 — Figure S2. Patients by stage CKD, age and sex. [file 1471-2369-14-24-S2.tiff]
